# RETRACTION: Tanshinone I attenuates the malignant biological properties of ovarian cancer by inducing apoptosis and autophagy via the inactivation of PI3K/AKT/mTOR pathway

**DOI:** 10.1111/cpr.13768

**Published:** 2024-10-23

## Abstract

ZhouJ. , JiangY.‐y. , ChenH. , WuY.‐c. , and ZhangL. , “Tanshinone I Attenuates the Malignant Biological Properties of Ovarian Cancer by Inducing Apoptosis and Autophagy via the Inactivation of PI3K/AKT/mTOR Pathway,” Cell Proliferation 53, no. 2 (2020): e12739. 10.1111/cpr.12739.31820522 PMC7046305

The above article, published online on 09 December 2019, in Wiley Online Library (wileyonlinelibrary.com), and has been retracted by agreement between the journal Deputy Editor, Yunfeng Lin; and John Wiley & Sons Ltd. A third party contacted the publisher to report that duplicated images had been detected in multiple different articles by different author groups, each of which described different experimental conditions. The image duplications are listed as follows: Images in Figure 1C were duplicated in Wang et al. 2019 (https://doi.org/10.2147/OTT.S221161), which was submitted and published prior to the publication of this article in *Cell Proliferation.* In addition, duplicate images from Figures 1C, 1D, 2C, 3B, 3D, 4C, 4D, and 5D were detected in many other articles by different author groups that had been published subsequent to this article.

The authors responded to an inquiry by the publisher regarding these concerns and shared what were labeled as original images for Figures 1–6 but did not share the original images for the cell proliferation assays that had been used in Figure 1. The editors reviewed this evidence and found that it did not properly explain the duplications across different articles. The retraction has been agreed to because the duplications of images across different articles fundamentally compromises the conclusions and results presented in the article. The authors disagree with the retraction.

J. Zhou , Y.‐y. Jiang , H. Chen , Y.‐c. Wu , and L. Zhang , “Tanshinone I Attenuates the Malignant Biological Properties of Ovarian Cancer by Inducing Apoptosis and Autophagy via the Inactivation of PI3K/AKT/mTOR Pathway,” Cell Proliferation 53, no. 2 (2020): e12739. 10.1111/cpr.12739.31820522 PMC7046305

The above article, published online on 09 December 2019, in Wiley Online Library (wileyonlinelibrary.com), and has been retracted by agreement between the journal Deputy Editor, Yunfeng Lin; and John Wiley & Sons Ltd. A third party contacted the publisher to report that duplicated images had been detected in multiple different articles by different author groups, each of which described different experimental conditions. The image duplications are listed as follows: Images in Figure 1C were duplicated in Wang et al. 2019 (https://doi.org/10.2147/OTT.S221161), which was submitted and published prior to the publication of this article in *Cell Proliferation.* In addition, duplicate images from Figures 1C, 1D, 2C, 3B, 3D, 4C, 4D, and 5D were detected in many other articles by different author groups that had been published subsequent to this article.

The authors responded to an inquiry by the publisher regarding these concerns and shared what were labeled as original images for Figures 1–6 but did not share the original images for the cell proliferation assays that had been used in Figure 1. The editors reviewed this evidence and found that it did not properly explain the duplications across different articles. The retraction has been agreed to because the duplications of images across different articles fundamentally compromises the conclusions and results presented in the article. The authors disagree with the retraction.

